# Cutaneous Tethering and Facial Dimpling As the Primary Manifestations of a Chronic Maxillary Odontogenic Cutaneous Sinus Tract: A Case Report and Focused Review of the Morphologic Evolution of Chronic Odontogenic Fistulas

**DOI:** 10.7759/cureus.117397

**Published:** 2026-10-03

**Authors:** Syed Immad Shah, Marij W Janjua, Josh Samoohi, Delvin Swift, Paul Baker

**Affiliations:** 1 Oral and Maxillofacial Surgery, New York University, New York City, USA

**Keywords:** chronic periapical infection, cutaneous lesion, facial dimpling, facial scar, fibrosis, maxillary premolar, odontogenic cutaneous sinus tract, odontogenic fistula, skin tethering

## Abstract

Odontogenic cutaneous sinus tracts (OCSTs) are uncommon manifestations of chronic odontogenic infection in which inflammatory drainage extends from a periapical source through cortical bone and adjacent soft tissues before reaching the skin surface. Although these lesions are well recognized for their ability to mimic dermatologic, infectious, and neoplastic conditions, comparatively little attention has been devoted to the morphologic evolution of chronic lesions into fibrotic, tethered, and dimpled facial defects. Recognition of these subtle physical examination findings may facilitate earlier diagnosis before unnecessary medical or surgical intervention.

We report the case of a 32-year-old male presenting with a painless indurated dimpled lesion involving the left cheek. Extraoral examination demonstrated localized cutaneous tethering with a small central cutaneous opening, while intraoral palpation revealed a firm ropy tract extending through the buccal vestibule adjacent to tooth #12. Periapical radiography demonstrated a chronic periapical radiolucency associated with tooth #12, and gutta-percha tracing confirmed direct communication between the cutaneous lesion and the apex of the involved tooth. The patient was diagnosed with a chronic maxillary OCST secondary to pulpal necrosis and chronic apical infection. Endodontic therapy was recommended; however, the patient was subsequently lost to follow-up before definitive treatment could be completed.

This case highlights cutaneous tethering and facial dimpling as underrecognized manifestations of longstanding odontogenic infection. Awareness of these physical examination findings may improve diagnostic accuracy, facilitate earlier recognition of chronic odontogenic disease, and reduce unnecessary dermatologic and surgical procedures.

## Introduction

Odontogenic cutaneous sinus tracts (OCSTs) represent an uncommon extraoral manifestation of chronic pulpal necrosis and periapical infection in which inflammatory drainage extends through cortical bone and adjacent fascial planes before establishing communication with the skin surface [1-3]. Although odontogenic infections most commonly drain intraorally through the buccal or lingual vestibule, persistent infections may occasionally follow the path of least resistance and present as chronic facial nodules, draining sinuses, cystic lesions, or localized cutaneous defects [1,2]. Because many patients experience little or no dental discomfort following chronic decompression of the infectious process, these lesions frequently present first to dermatologists, family physicians, plastic surgeons, otolaryngologists, or general surgeons before a dental etiology is considered [1,2,4].

Large clinical series have demonstrated that OCSTs are frequently misdiagnosed because of their highly variable appearance and the absence of significant odontogenic symptoms [1,2]. Reported clinical mimics include epidermoid cysts, furuncles, pyogenic granulomas, salivary gland pathology, chronic osteomyelitis, actinomycosis, branchial anomalies, basal cell carcinoma, squamous cell carcinoma, and metastatic disease [1,2,5-7]. Failure to recognize the underlying odontogenic etiology may result in prolonged antibiotic therapy, repeated incision and drainage procedures, unnecessary biopsies, cosmetic excisions, and delayed definitive treatment [1,2,4].

While the diagnostic challenges associated with OCSTs have been well documented [1-4], comparatively little attention has been devoted to the morphologic evolution of chronic lesions. As inflammatory drainage persists over months or years, repetitive cycles of tissue injury and repair may result in progressive collagen deposition, fibrosis, and adhesion between the dermis and deeper soft tissues [8-10]. Consequently, some longstanding lesions lose their characteristic draining appearance and instead present as areas of facial skin retraction, tethering, or dimpling with minimal active drainage. These subtle physical examination findings may resemble healed scars, localized fibrosis, acneiform scarring, or other benign cutaneous abnormalities rather than manifestations of persistent odontogenic infection [2,8].

Recognition of these chronic morphologic changes is clinically important because they may represent the only external evidence of an underlying periapical infection. Careful inspection combined with palpation may reveal a firm cord-like tract extending toward the involved tooth with fixation of the overlying skin, providing an important diagnostic clue even when drainage is absent [2,4]. Despite their potential diagnostic value, cutaneous tethering and facial dimpling have received relatively limited discussion within the existing literature and are often mentioned only briefly as possible clinical findings without detailed consideration of their pathogenesis or physical examination characteristics.

We present the case of a chronic maxillary OCST associated with tooth #12 in which the predominant clinical finding was localized facial dimpling with dermal tethering and a palpable fibrotic tract rather than an actively draining sinus. The case highlights an underrecognized manifestation of longstanding odontogenic infection and reviews the morphologic evolution responsible for these characteristic physical examination findings.

## Case presentation

Patient information

A 32-year-old male presented to the New York University College of Dentistry Oral and Maxillofacial Surgery Clinic on Day 1 for evaluation of a chronic lesion involving the left cheek. The patient reported experiencing pain involving the maxillary left dentition approximately two years before presentation, which he self-managed with topical medication without seeking definitive dental treatment. Approximately seven months before presentation, he noticed gradual development of a small depression within the skin of the left cheek that progressively became indurated. He denied facial swelling, fever, purulent drainage, trismus, dysphagia, constitutional symptoms, or pain at the time of presentation.

Clinical examination

Extraoral examination demonstrated a localized indurated cutaneous depression involving the left cheek with characteristic facial dimpling and tethering of the overlying skin (Figures 1A-1B). A small central cutaneous opening was identified within the area of fibrosis; however, active purulent drainage was absent. The surrounding skin demonstrated mild fibrosis without significant erythema.

**Figure 1:**
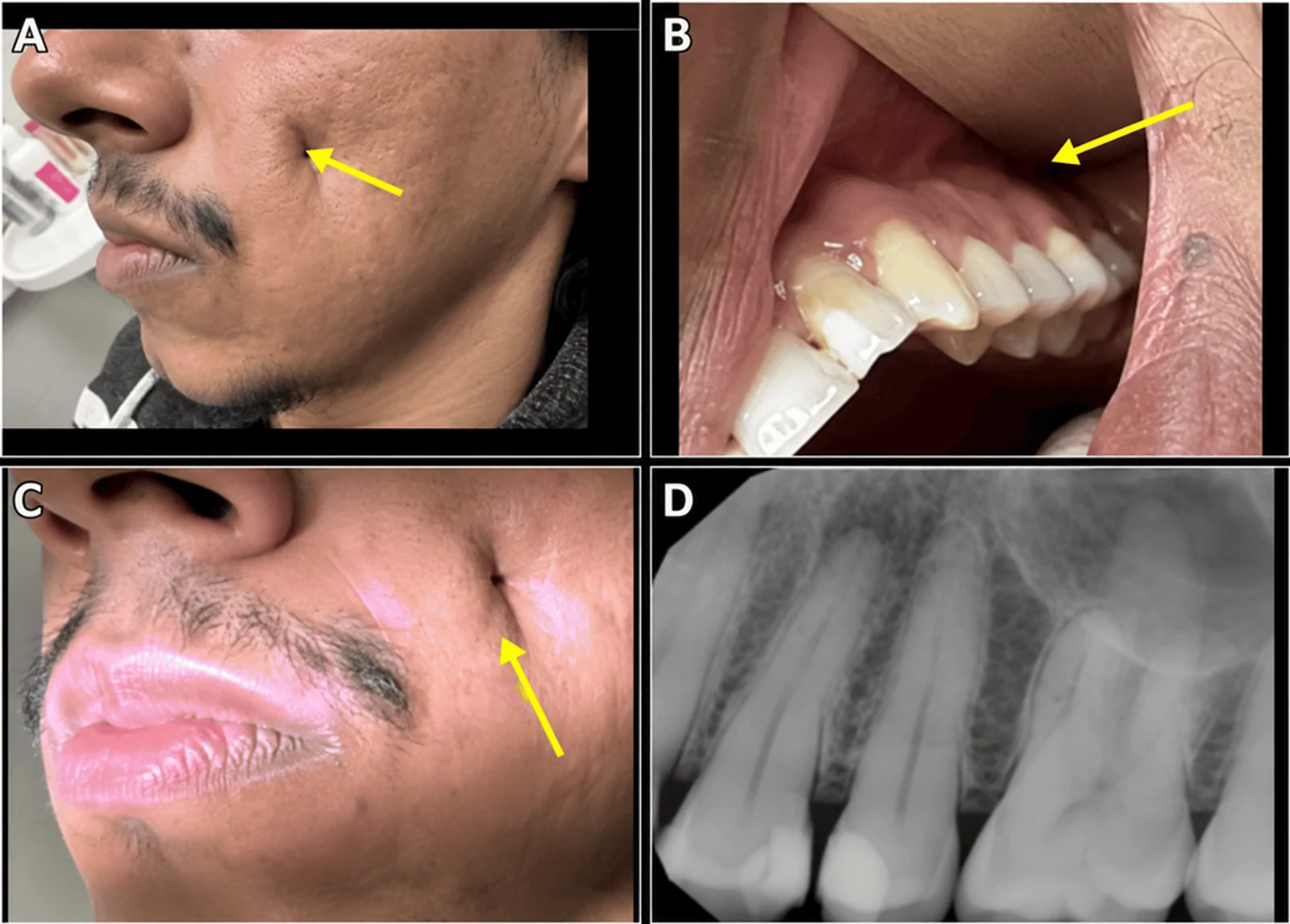
Clinical and radiographic features of a chronic maxillary odontogenic cutaneous sinus tract. (A) Initial clinical presentation demonstrating a cutaneous sinus tract with central dimpling on the left cheek. (B) Intraoral examination revealing a corresponding maxillary buccal mucosal dimple in the vestibule apical to the premolar region, consistent with the intraoral opening of the sinus tract. (C) Extraoral photograph demonstrating resolution of the cutaneous drainage with persistence of residual skin dimpling and scar formation. (D) Panoramic radiograph demonstrating the associated maxillary odontogenic lesion and sinus involvement.

Intraoral examination revealed a palpable firm ropy tract extending through the left buccal vestibule adjacent to tooth #12. Palpation reproduced fixation of the overlying skin, demonstrating continuity between the intraoral inflammatory tract and the extraoral cutaneous lesion. No significant vestibular swelling or fluctuance was present.

Radiographic findings

Periapical radiographic examination demonstrated a well-circumscribed periapical radiolucency associated with the apex of tooth #12 (Figure 1C), consistent with chronic periapical inflammatory disease. Gutta-percha was gently inserted through the cutaneous opening, and subsequent radiographic imaging demonstrated direct communication between the sinus tract and the apex of tooth #12 (Figure 1D), confirming the odontogenic origin of the lesion.

Diagnosis

Chronic maxillary OCST secondary to pulpal necrosis and chronic periapical infection involving tooth #12.

Management

The patient was informed that tooth #12 was considered restorable and was referred to the NYU College of Dentistry Endodontic Clinic for definitive treatment. On Day 2, pulpal vitality testing established a diagnosis of necrotic pulp with chronic apical abscess. Nonsurgical root canal therapy was recommended to eliminate the odontogenic source of infection. Despite counseling regarding the expected resolution of the cutaneous lesion following definitive endodontic treatment, the patient did not return to initiate therapy. Consequently, long-term clinical resolution of the facial lesion could not be documented.

## Discussion

OCSTs represent an uncommon manifestation of chronic periapical infection in which inflammatory drainage extends through cortical bone and adjacent soft tissues before establishing communication with the skin surface [1,2]. Although the diagnostic challenges associated with these lesions have been well described, considerably less attention has been devoted to the progressive morphologic changes that occur as chronic inflammation persists over time. Rather than presenting as actively draining sinus tracts, longstanding OCSTs may evolve into indurated, fibrotic lesions characterized by dermal tethering, facial dimpling, and localized cutaneous retraction. The present case illustrates this less frequently emphasized stage of disease progression, in which chronic fibrosis became the dominant clinical feature despite minimal active drainage.

The evolution of a chronic OCST reflects the normal biologic response to persistent inflammation. Chronic pulpal necrosis results in continuous periapical infection with gradual cortical perforation and extension of inflammatory exudate along the path of least resistance through adjacent fascial planes [3,4]. As drainage becomes established, repeated cycles of tissue injury and repair stimulate fibroblast proliferation, collagen deposition, and extracellular matrix remodeling within the sinus tract [8-10]. Over time, this progressive fibrosis may tether the dermis to deeper soft tissues, producing the characteristic cutaneous depression and skin fixation observed in the present patient. Rather than representing an acute infectious process, facial dimpling may therefore signify a mature sinus tract that has undergone chronic structural remodeling.

These morphologic changes have important diagnostic implications. While actively draining lesions are more readily recognized as sinus tracts, chronically fibrotic lesions may resemble acne scars, traumatic scars, localized fibrosis, healed cysts, or other benign cutaneous abnormalities. In the present case, the absence of pain, facial swelling, and active purulent drainage shifted the clinical appearance away from an infectious process and toward a seemingly innocuous cutaneous defect. Careful inspection alone, however, was insufficient to establish the diagnosis. Palpation demonstrated a firm, ropy tract extending from the buccal vestibule toward the cutaneous depression with fixation of the overlying skin, providing an important clinical clue to the underlying odontogenic origin. Although cutaneous dimpling has occasionally been described within the OCST literature, detailed discussion of dermal tethering and its underlying pathophysiology remains limited [2,5].

The anatomic location of the offending tooth also contributes to the educational value of this case. Most reported OCSTs arise from mandibular teeth, particularly the mandibular molars, because infection commonly perforates the thinner buccal cortex inferior to the attachments of the mentalis, buccinator, or mylohyoid muscles before extending toward the skin surface [3,6]. In contrast, infections originating from maxillary teeth more frequently drain intraorally because of differences in cortical anatomy and muscle attachments. Extraoral drainage from maxillary premolars therefore represents a less common pattern of disease progression. In the present case, chronic infection associated with tooth #12 tracked through the buccal soft tissues before presenting as a localized tethered lesion of the cheek, illustrating the influence of local anatomic relationships on sinus tract formation.

Accurate diagnosis depended upon correlation of the clinical examination with radiographic findings. Periapical radiography demonstrated a chronic periapical radiolucency associated with tooth #12, while gutta-percha tracing established direct communication between the cutaneous opening and the involved tooth. Despite the increasing availability of cone beam computed tomography, gutta-percha tracing remains one of the most reliable and cost-effective techniques for confirming the odontogenic origin of a draining sinus tract when a patent cutaneous opening is present [4,7]. In cases where drainage is intermittent or the tract cannot be cannulated, CBCT may provide additional information regarding cortical perforation, sinus tract direction, and adjacent anatomic structures [3].

An additional teaching point illustrated by this case is the discrepancy between disease chronicity and symptom severity. The patient reported dental pain approximately two years before presentation but remained asymptomatic after development of the cutaneous lesion. This clinical course is consistent with previous reports demonstrating that chronic sinus tract formation decompresses periapical infection, reducing intramedullary pressure and eliminating the acute pain typically associated with odontogenic abscesses [1,2]. Consequently, patients frequently underestimate the significance of the lesion and may delay seeking definitive dental care. In the present case, although appropriate endodontic therapy was recommended following confirmation of pulpal necrosis and chronic apical abscess, the patient did not return to initiate treatment. Persistent infection therefore likely remained despite establishment of the diagnosis, highlighting the importance of patient education regarding the need to eliminate the primary odontogenic source rather than simply monitoring the cutaneous lesion.

The prognosis of OCSTs is generally excellent once the underlying dental infection has been eliminated through endodontic therapy or extraction [2,4,6]. Most lesions resolve spontaneously without requiring surgical excision of the sinus tract. However, prolonged disease duration may result in persistent dermal fibrosis and residual cosmetic deformity despite successful eradication of the infection [8,9]. Early recognition of subtle physical examination findings such as facial dimpling, cutaneous tethering, and palpable fibrotic tracts may therefore facilitate earlier intervention before irreversible scar formation develops.

This case contributes to the existing literature by emphasizing an underrecognized morphologic presentation of chronic OCST rather than its differential diagnosis alone. While previous reports have highlighted the ability of these lesions to mimic dermatologic disease and malignancy, the present case demonstrates that longstanding odontogenic infection may instead manifest primarily as a fibrotic tethered facial depression with minimal evidence of active infection. Recognition of these chronic physical examination findings broadens the clinical spectrum of OCSTs and reinforces the importance of including odontogenic disease in the differential diagnosis of unexplained facial skin retraction, dimpling, or localized fibrosis.

## Conclusions

This case demonstrates the potential for chronic odontogenic infections to mimic dermatologic conditions and highlights the importance of correlating clinical findings with dental radiographic evaluation. The odontogenic origin of these lesions is often overlooked by both patients and healthcare providers, leading patients to seek evaluation from multiple medical specialties before ultimately being diagnosed by a dentist. Chronic OCSTs may present as tethered or dimpled facial lesions with minimal drainage or dental symptoms. Palpation of a cord-like tract and targeted dental radiography can help identify the odontogenic source. Early recognition of the underlying dental etiology of these persistent cutaneous manifestations can facilitate timely definitive treatment while preventing unnecessary delays, misdiagnoses, and procedures. Therefore, both medical and dental professionals should consider this uncommon presentation of OCSTs in the differential diagnosis of persistent cutaneous lesions to improve diagnostic accuracy and patient outcomes.
